# Management of chronic non-communicable diseases in Ghana: a qualitative study using the chronic care model

**DOI:** 10.1186/s12889-021-11170-4

**Published:** 2021-06-11

**Authors:** Hubert Amu, Eugene Kofuor Maafo Darteh, Elvis Enowbeyang Tarkang, Akwasi Kumi-Kyereme

**Affiliations:** 1 Department of Population and Behavioural Sciences, School of Public Health, University of Health and Allied Sciences, Hohoe, Ghana; 2 Department of Population and Health, University of Cape Coast, Cape Coast, Ghana

**Keywords:** Chronic disease, Disease management, Ghana, Health personnel, Patients, Qualitative research

## Abstract

**Background:**

While the burden and mortality from chronic non-communicable diseases (CNCDs) have reached epidemic proportions in sub-Saharan Africa (SSA), decision-makers and individuals still consider CNCDs to be infrequent and, therefore, do not pay the needed attention to their management. We, therefore, explored the practices and challenges associated with the management of CNCDs by patients and health professionals.

**Methods:**

This was a qualitative study among 82 CNCD patients and 30 health professionals. Face-to-face in-depth interviews were used in collecting data from the participants. Data collected were analysed using thematic analysis.

**Results:**

Experiences of health professionals regarding CNCD management practices involved general assessments such as education of patients, and specific practices based on type and stage of CNCDs presented. Patients’ experiences mainly centred on self-management practices which comprised self-restrictions, exercise, and the use of anthropometric equipment to monitor health status at home. Inadequate logistics, work-related stress due to heavy workload, poor utility supply, and financial incapability of patients to afford the cost of managing their conditions were challenges that militated against the effective management of CNCDs.

**Conclusions:**

A myriad of challenges inhibits the effective management of CNCDs. To accelerate progress towards meeting the Sustainable Development Goal 3 on reducing premature mortality from CNCDs, the Ghana Health Service and management of the respective hospitals should ensure improved utility supply, adequate staff motivation, and regular in-service training. A chronic care management policy should also be implemented in addition to the review of the country’s National Health Insurance Scheme (NHIS) by the Ministry of Health and the National Health Insurance Authority to cover the management of all CNCDs.

**Supplementary Information:**

The online version contains supplementary material available at 10.1186/s12889-021-11170-4.

## Background

Chronic non-communicable diseases (CNCDs) have become the heaviest burden to healthcare systems worldwide [1–3]. About 41 million people die of CNCDs each year, accounting for 71% of all global deaths [4]. While CNCDs are traditionally thought to be more prevalent in developed countries, the majority of the increase in these diseases globally is occurring in low- and middle-income countries (LMICs) [5]. Out of the 41 million deaths which are attributable to CNCDs each year, 85% are in LMICs [4]. The CNCD burden is particularly problematic for sub-Saharan Africa (SSA) since CNCDs are already a major cause of mortality in the sub-region [6]. CNCDs prevail in the sub-region at the backdrop that meeting the needs of the ever-increasing population in SSA is critical to achieving the Sustainable Development Goal (SDG) 3, which seeks to ensure healthy lives and promote well-being for all at all ages, and specifically, target 3.4, which seeks to reduce, by one third, premature mortality from CNCDs through prevention and treatment by the year 2030 [7].

In Ghana, the burden and mortality from CNCDs have achieved epidemic proportions [8]. It has been estimated that CNCDs are responsible for about 43% of deaths in the country [9]. Despite this finding, decision-makers and individuals still consider CNCDs as infrequent and as such, do not pay the needed attention to their management [8, 10]. While several studies have been conducted on CNCDs in the country [8, 11–21], there is a paucity of health facility-based qualitative empirical research on the management of CNCDs. The studies conducted were either review studies that drew conclusions based on other studies, used secondary data from the Wave 1 of the World Health Organisation’s Study on Global Ageing and Adult Health (SAGE) conducted from 2008 to 2009, or used primary data collected from participants. For most of the studies which used primary data, the participants were adults 18 years and above. One of the studies was partly done qualitatively using focus group discussions [21]. It was, however, conducted among lay people (described in the study as people who did not necessarily have a chronic disease) and focused on their views of the prevalence of various CNCDs (thus, the chronic diseases that they think were most prevalent in Ghana). A study by de-Graft Aikins et al. [15] showed that biomedical care and spiritual action are management options people in Ghana adopt in dealing with their CNCDs. Mosques, churches, and other religious institutions, for instance, played essential roles in the spiritual actions people take in managing their CNCDs. Management practices by health professionals are also essential in the biomedical management of CNCDs.

In August 2012, Ghana implemented a national policy for the prevention and control of CNCDs [22]. The policy seeks to reduce the incidence, prevalence, and exposure of people to CNCD risk, reduce morbidity associated with CNCDs, and improve the overall quality of life of persons living with CNCDs. It focuses on strategies such as primary prevention and clinical care including early detection, provision of treatment services, health system strengthening involving the training of health workers, and the development of human resource capacity. The policy, however, pays limited attention to the management of patients who already have the CNCDs. Given the paucity of literature on management practices of CNCDs by patients and health professionals in Ghana, our objective was to explore the experiences of patients and health professionals regarding their CNCD management practices and challenges at the Korle Bu Teaching Hospital (KBTH) and Komfo Anokye Teaching Hospital (KATH). Our study contributes to the discourse on the management practices and challenges of CNCD in Ghana, as the country struggles to manage the double burden of CNCDs and infectious diseases [16]. With an increased knowledge on the challenges juxtaposed to our recommendations in addressing them, policymakers would be better informed and equipped to develop innovative policy interventions or expand existing ones that seek to improve the management of CNCDs.

## Theoretical issues

The chronic care model (CCM) underpinned our study. The original CCM was developed by the MacColl Institute for Healthcare Innovation at Group Health Cooperative in 1992 [23]. The CCM is an evidence-based multifaceted framework aimed at enhancing chronic care by providing an organised approach to practice transformation [24]. It was, however, developed in the context of high-income countries, raising concerns regarding its applicability to LMICs like Ghana. Several studies have, therefore, adapted the CCM to fit LMIC contexts. We followed the adapted version of the CCM by Lall et al. [25] to develop a framework for our study based on the themes we realised. The key tenets of the CCM are the health systems, decision support, clinical information systems for individual care plan, self-management support, community resources and policies, and delivery system design [24].

**Fig. 1:**
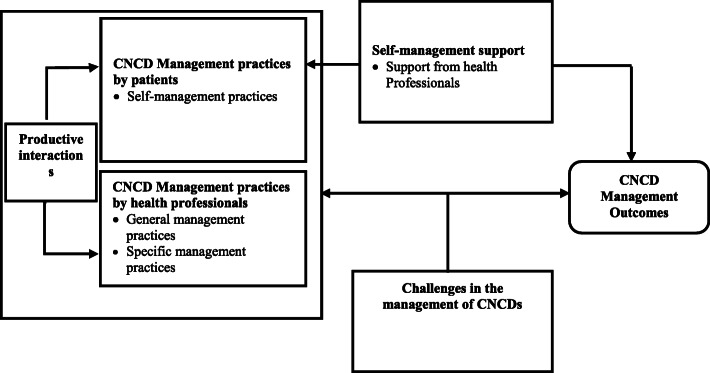
Conceptual framework

While the health systems tenet of the original model focused on creating a culture, organisation, and mechanisms that promote high-quality and safe healthcare, Lall et al. [25] adapted this to become a leadership that is motivated to handle errors systematically and make sustained improvements. Regarding self-management support, while the original model focused on empowering and preparing patients to manage their conditions, Lall et al. [25] emphasised the centrality of the patient in their healthcare and also highlighted the actual self-management practices of the patients, which is essential in achieving desirable management outcomes. Decision support in the original model also sought to promote clinical care which is consistent with scientific evidence and preferences of patients. The adapted version then incorporated the use of evidence-based guidance through health worker education of patients on managing their CNCDs. The community recourses and policies tenet of the original model was also adapted by Lall et al. [25] to incorporate partnerships that seek to support and fill gaps in healthcare, with community organisations. In their review of the literature to adapt the CCM, Lall et al. [25] recognised that in LMICs, a myriad of challenges militated against the effective management of CNCDs. These include the non-availability or inadequacy of health personnel, equipment, medicines, and laboratory supplies. Figure 1 presents the conceptual framework we created based on Lall et al.’s [25] adaptation of the CCM in line with our themes which were mainly a priori in nature.

## Methods

The consolidated criteria for reporting qualitative research (COREQ) [26] was adopted in reporting this research (See Additional file 1).

### Setting

Our study was conducted at KBTH and KATH. We chose these two hospitals because they are the top two hospitals in Ghana and receive both primary and referral cases from all over the country [27]. KATH typically receives cases from the northern half of the country [28] while KBTH mainly receives cases from the southern half of the country [29, 30].

Established on October 9, 1923, KBTH grew from an initial 200-bed capacity to over 2000 [31]. It is located in Accra, the capital of Ghana, and is presently the third-largest hospital in Africa as well as the leading national referral centre in Ghana. The hospital currently has an average daily attendance of 1500 patients and about 250 admissions [31]. Established in 1954, KATH is the second-largest hospital in Ghana after KBTH. It is located in Kumasi, the capital of the Ashanti Region [32]. KATH currently has a bed capacity of 1000, from an initial 500 beds [32].

The organisation of healthcare is based primarily on departments/centres established at both KATH and KBTH. The major departments/centres at the hospitals comprise child health, central laboratory, medical and surgical, radiotherapy, obstetrics and gynaecology, accidents and emergency centre, dietherapy, physiotherapy, eye centre, pathology, psychiatry, radiology, pharmacy, finance, engineering, and general administration, biostatistics, and anaesthesia [30, 32]. Departmental/centre heads oversee daily management and healthcare delivery at the various departments. At the top hierarchy of each hospital, however, is a chief executive officer (CEO) who is assisted by directors of pharmacy, medical affairs, nursing services, administration, finance, general services, and human resources to oversee the overall administration of the hospitals. Data collection for our study was conducted at the medical and surgical, radiotherapy, physiotherapy, and eye centre where the respective CNCDs are mainly managed.

### Study design

We used a qualitative case study design to explore the experiences of patients and health professionals on their CNCD management practices and challenges [33]. The adoption of a qualitative approach to explore the practices and challenges associated with the management of CNCDs enabled us to obtain in-depth information from the patients and health professionals regarding their experiences with the management of CNCDs at KATH and KBTH.

### Study population

The primary target population for this study comprised people living with CNCDs, who had experiences accessing services related to their diseases from KBTH and KATH. To obtain expert views and experiences on CNCD management as the study was health facility-based, health professionals who directly manage the CNCDs were also targeted. They included medical doctors, nurses, physiotherapists, and optometrists. To be recruited, however, the health professionals needed to have worked for at least 6 months at the hospitals and managing the condition for which they were interviewed. This was to ensure that only health professionals who had considerable experience managing the conditions were recruited.

### Sampling

Purposive and accidental sampling procedures were adopted in recruiting participants for the study. Maximum variation sampling was adopted to ensure that participants were recruited for all the diseases (diabetes, cancer [breast, prostate, cervical, leukaemia, eye, and lung], chronic kidney disease [CKD], chronic obstructive pulmonary disease [COPD], asthma, hypertension, sickle cell disease, glaucoma, and stroke). This resulted in the sampling of 41 patients and 15 health professionals at each of the hospitals. The size of the sample included in the study was based on data saturation [34]. This was because no clear sampling framework existed to chose participants from as new cases kept being reported while referred cases also cease reporting to the hospitals once the period of referral was over and they resume management of the CNCDs at their localised health facilities. Details of the sampling procedure are presented in Table 1.

**Table 1:** Sampling of participants

Situation	Sampling procedure	Recruitment Strategy/ Inclusion Criteria
Out-patient units with designated clinic days for patients with a particular CNCD	Accidental sampling	Exit strategy was used to recruit patients after receiving care
Out-patient units where several health conditions were presented including those that the study was not interested in	Purposive sampling	Exit strategy was used to recruit patients after receiving care
In-patient units/departments which had several patients	Purposive sampling	• The nurse in charge helped to identify patients with CNCDs of interest to the study using patients’ registers • Patients who were active/stable, could communicate clearly, and not in pain/discomfort were recruited
Patients with co-morbidities	Purposive	• Patients with comorbidities of interest to the study were recruited for all the CNCDs they had. • Patients with comorbidities of interest to the study were recruited for all the CNCDs they had
Health Professionals	Purposive	• Only health professionals managing CNCDs of interest to the study were recruited • Health professionals who had worked in the respective departments/unit for at least 6 months, managing the respective CNCDs were recruited

Twenty-four patients and 14 health professionals refused to participate in the study when approached by the research team. While the patients indicated being in a hurry to go back home, the health professionals mentioned that they were too busy to make time for the interviews. One health professional’s interview could not be completed, as they had to leave the interview to attend to an emergency. The uncompleted interview was, thus, expunged.

### Research team

Data were collected by the first author (male) and three field assistants (one female and two males) who were all master’s degree graduates and had considerable experience in the conduct of qualitative interviews. The assistants were trained for 2 days to acquaint them with the research instruments.

### Data collection

In-depth interviews were used in collecting data from the patients and health professionals through face-to-face interactions at the two hospitals. Even though the data were collected at the hospitals, interviews were conducted at distances/locations where others could not listen to the discussions. All interviews with health professionals were conducted in English. Twenty-two of the patients’ interviews were conducted in Ghanaian languages (Twi and Ga) while the rest were conducted in English. To ensure that the use of the local languages did not affect the quality of data collected, the training organised for the assistants also focused on interpretations of the instruments in the languages to ensure that the right questions were asked. While patients’ interviews generally lasted for about 35 min, the health professionals’ interviews took about 45 min. There were no repeat interviews, as all the face-to-face interactions were single time interviews.

Data collection for the study lasted from January to May 2019. Hand-written notes (with pens and notebooks) and audio recorders were used to record the interviews. The use of both audio recorders and hand-written notes was to ensure that interviews were not halted should any of the equipment break down during the interview process. No relationships were established with the participants before the data collection. During the interviews, however, they were informed that the research formed part of the first author’s PhD requirements. The participants were interviewed with two in-depth interview guides (IDIs), one for patients and the other for health professionals. Both instruments had three sections; A–C. Section A focused on the background characteristics of the participants (eg., sex, age, marital status, religion, ethnicity educational level, and place of residence). Section B explored the management practices of CNCDs while section C was based on the challenges associated with managing CNCDs. Details of the instruments are contained in Additional file 2. Probes were used to obtain detailed information from the participants whenever necessary. Before the main data collection, the instruments were pre-tested among three health professionals and five CNCD patients at a municipal hospital in the Volta Region of Ghana. The pre-testing led to the reframing of two questions in the patients’ interview guide and one question in the health professionals’ interview guide to make them clearer.

### Trustworthiness

Trustworthiness of qualitative research according to Korstjens and Moser [35] entails credibility, transferability, dependability, and confirmability. Credibility is concerned with the aspect of truth-value and is measured with strategies including triangulation. In the current study, data triangulation was the main type of triangulation adopted. With this, findings from KBTH and KATH were realized as communicating similar issues. They were, thus, presented homogenously without comparisons between the two sites. Triangulations were also done between patients and health professionals’ results. The themes that were triangulated were self-management and challenges of patients in the management of their conditions. The fact that two researchers independently generated the codes also ensured investigator triangulation where the coders had to compare their codebooks and agree on common codes and themes.

Transferability has to do with a thick description. This involves the description of not just the experiences and behaviours of study participants, but also a detailed account is given on the context in which the study was conducted. This ensures that the experiences and behaviours become meaningful to an outsider [35]. In the current research, transferability was ensured by describing the study setting, providing the sample size and sampling procedure used, and describing the socio-demographics of the study participants in addition to the CNCDs of patients interviewed. Transferability was also ensured in the present study by providing the thematic table and the fact that samples of the interview guides used in collecting data from the participants have been provided as additional files.

Dependability and confirmability focus on the audit trail [35]. The audit trail is about transparency in the description of the research processes from the beginning of a study to the development and reporting of the results. In the current study, the audit trail was ensured by documenting the entire research process from the background to the conclusion section. Regarding analyses, the thematic table has been provided. The interpretations of the data were also derived from the data collected and not based on the researcher’s own preferences and viewpoints, which was made clear by providing a sub-section on positionality (reflexivity).

### Reflexivity

As researchers in population and health, we wanted to understand the issue of CNCDs from a management perspective. This is because we had read several studies on measures to prevent such diseases, including healthy eating, exercising, and taking enough rest. However, the issue of managing CNCDs was usually not extensively covered. We also became concerned that even though there has been much discussion on preventing CNCDs through the measures enumerated, the diseases are, however, still on the ascendancy in Ghana. We, therefore, wanted to understand how the diseases are managed in terms of the practices involved. This was because, we felt that if managed effectively, deaths that occur due to the CNCDs would be low or entirely averted. On the contrary, they kept increasing.

As lecturers of Public Health, we teach courses on change interventions for chronic diseases where students are introduced to the aetiology, causes/risk factors, prevention, and treatment/management of CNCDs. We always had difficulty identifying scholarly publications focusing on the management of the conditions particularly in Ghana with an emphasis on policy interventions, as we realised that there was no policy dedicated to the management of CNCDs in Ghana.

Moreover, while at the hospital to receive care as patients, there were usually other patients, particularly those with CNCDs who complained about the care they were receiving from the health professionals. Some, for instance, mentioned that whenever they wanted to understand the extent of their conditions, the health professionals were not forthcoming with responsive answers but rather became offended. Overall, as individuals, we feel very empathetic towards persons living with CNCDs, as these are conditions which sometimes were acquired through no fault of the victims but which they have to live with for the rest of their lives and some even die prematurely from them. We, therefore, wanted to understand further, how the diseases are managed and the vicissitudes involved in doing so.

### Ethical issues

Ethical clearance was obtained from the University of Cape Coast Institutional Review Board (IRB) (UCCIRB/CHLS/2018/07) and the Institutional Review Boards of KATH (KATH: RD/CR18/251 & KNUST: CHRPE/AP/023/19) and KBTH (KBTH-STC 000124/2018 and KBTH-IRB/000124/2018). Permission was also sought from department/centre heads at the hospitals before data were collected. Informed consent was obtained from participants before including them in the study. This was achieved by giving them informed consent forms to sign/thumbprint. Confidentiality was ensured by using pseudonyms instead of the real names and other characteristics of the participants. Audio recordings were locked with a password-protected computer programme called ‘my lockbox’*.* Notes taken were typed and the soft copies equally locked in ‘my lockbox’*.*

### Data analysis

Data collected were transcribed, proofread, and prepared for analysis by combining all the transcripts into two Microsoft Word files, one for health professionals and the other for patients. Interviews not conducted in English were transcribed into English. To minimise errors, transcribed interviews were compared with notes taken during interviews and proofread while listening to the audio recordings. The transcripts were, however, not returned to the participants for correction, as it was going to be either impossible or take a prolonged period to ensure all 112 participants corrected the transcripts. This is because some of the patients lived in other regions of the country before accessing care at the hospitals and were not going to return for reviews at all or any time soon.

Data collected were analysed using reflexive thematic analysis [36]. With this, the transcriptions were read and re-read to ensure familiarity with the data. HA mainly did the coding and generation of themes. To ensure inter-coder reliability, AKK also did confirmatory coding of the data independently. First, a codebook was created. With this, preliminary codes were identified with corresponding frequencies from the responses of the participants. Codes were then collated and sorted based on their shared patterns to form sub-themes and, subsequently, main themes. Where needed, themes were combined, separated, or discarded to define a pattern of shared meaning projected by a central idea. After this, the themes were refined and defined by providing names and clear working definitions capturing the essence of each theme. Finally, descriptive narratives of the themes together with analytic narrative and data extracts were used to contextualise the analysis. HA and AKK compared their codebooks to agree on final codes and themes. The coding was done using ATLAS-ti version 7.5.7 by ATLAS.ti GmbH, Berlin. Statements of the respondents are presented as quotes to illustrate the findings. Frequency tables are used to present the socio-demographic characteristics of the study participants and the CNCDs of patients.

## Results

We present the results of this study based on the themes from the analysis conducted. The background characteristics of the participants are also presented.

### Background characteristics of study participants

Table 2 presents the background characteristics of the health professionals. Fifty per cent were in their 30s. The majority were married (66.7%), Christians (93.3%), and nurses (66.7%). The comparative majority had also worked for 1–5 years in their respective professions (Table 2).

**Table 2:** Background characteristics of health professionals

Variable	KATH			KBTH			BOTH HOSPITALS		
	Male%	Female%	Total%(***n*** = 15)	Male%	Female%	Total %(***n*** = 15)	Male%	Female%	Total%(***n*** = 30)
**Age**									
20–29	13.3	20.0	33.3	–	26.7	26.7	6.7	23.3	30.0
30–39	20.0	33.3	53.3	6.7	40.0	46.7	13.3	36.7	50.0
40–49	6.7	6.7	13.4	6.7	6.7	13.3	6.7	6.7	13.3
50–59	–	–	–	–	13.3	13.3	–	6.7	6.7
**Level of formal education**									
Diploma	20.0	13.3	33.3	–	6.7	6.7	10.0	10.0	20.0
1st degree	13.3	46.7	60.0	–	79.9	79.9	6.7	63.3	70.0
2nd degree	6.7	–	6.7	6.7	–	6.7	6.7	–	6.7
3rd degree	–	–	–	6.7	–	6.7	3.3	–	3.3
**Marital status**									
Never married	26.7	13.3	40.0	–	20.0	20.0	13.3	16.7	30.0
Married	6.7	46.6	53.3	13.3	66.7	80.0	10.0	56.7	66.7
Divorced	–	–	–	–	–	–	–	–	–
Widowed	–	6.7	6.7	–	–	–	–	3.3	3.3
**Religion**									
Christianity	33.3	60.0	93.3	13.3	80.0	93.3	23.3	95.5	93.3
Islam	6.7	–	6.7	–	6.7	6.7	3.3	3.3	6.6
**Ethnicity**									
Mole-Dagbani	6.7	–	6.7	–	–	–	3.3	–	3.3
Ewe	6.7	–	6.7	–	6.7	6.7	3.3	3.3	6.3
Akan	26.6	60.0	86.6	13.3	66.7	80	20.0	63.3	83.3
Ga/Dangme^gbani^	–	–	–	–	13.3	13.3	–	6.7	6.7
**Occupation**									
Medical doctor	–	6.7	6.7	13.3	–	13.3	6.7	3.3	10.0
Nurse	20.0	53.3	73.3	–	60.0	60.0	10.0	56.7	66.7
Optometrist	–	–	–	–	6.7.0	6.7	–	3.3	3.3
Physiotherapist	13.3	6.7	20.0	–	20.0	20.0	6.7	13.3	20.0
**Duration of practice (In years)**									
1–5	20.0	33.3	53.3	–	40.0	40.0	10.0	36.7	46.7
6–10	20.0	20.0	40.0	13.3	26.7	40.0	16.7	23.3	40.0
11+	–	6.7	6.7	–	20.0	20.0	–	13.3	13.3

Table 3 presents the background characteristics of patients. About 51% were 60 years and above. About 45% of the patients had SHS/O’level/A’ level of education. The majority (86.6%) were Christians and more than 64% were married. By ethnicity, more than 53% were Akans. The majority (90.3%) of patients at KATH were from the northern part of the country while most patients at KBTH (95.2%) were from the southern part. Regarding the CNCDs of patients, we realised that more than 25% of the patients had comorbidities. Hypertension was present in almost all the comorbid cases recorded (See Table 3).

**Table 3:** Background characteristics of patients

Background characteristic	KATH			KBTH			BOTH HOSPITALS		
	Male%	Female%	Total%(***n*** = 41)	Male%	Female%	Total%(***n*** = 41)	Male%	Female%	Total%(***n*** = 82)
**Age**									
20–29	4.9	9.8	14.7	4.9	4.9	9.8	4.9	7.3	12.2
30–39	4.9)	7.3	12.2	–	–	–	2.4	3.7	6.2
40–49	4.9	4.9	9.8	7.3	4.9	12.2	6.1	4.9	10.9
50–59	9.8	9.8	19.6	9.8	9.8	19.6	9.8	9.8	19.5
60+	14.5	29.2	43.7	26.7	31.7	58.4	20.7	30.5	51.2
**Level of formal education**									
No formal education	2.3	9.8	12.1	2.4	4.9	7.3	2.4	7.3	9.8
Primary	–	9.8	9.8	–	2.4	2.4	–	6.1	6.1
Middle school/JHS	17.1	14.6	31.7	–	4.9	4.9	8.5	9.8	18.3
SHS/O’level/A’level	14.6	17.1	31.7	21.9	36.7	58.6	18.3	26.8	45.1
Tertiary	4.9	9.8	14.7	21.9	4.9	26.8	13.4	7.3	20.7
**Marital status**									
Never married	2.4	12.2	14.6	4.9	7.3	12.2	3.7	9.8	13.4
Married	34.1	19.6	53.7	41.5	34.1	75.1	37.8	26.8	64.7
Divorced	2.4	12.2	14.6	–	2.4	2.4	1.2	7.3	8.5
Widowed	–	17.1	17.1	–	9.8	9.8	–	13.4	13.4
**Religion**									
Christianity	36.6	53.7	90.3	36.6	46.3	82.9	36.6	50.0	86.6
Islam	2.4	7.3	9.7	7.3	9.8	17.1	4.9	8.5	13.4
**Occupation**									
Unemployed	2.4	14.6	17.0	4.9	17.1	22	3.7	15.9	19.5
Retired civil servant	2.4	7.3	9.7	14.7	2.4	17.1	8.5	4.9	13.4
Farmer	9.8	4.9	14.7	–	2.4	2.4	4.9	3.7	8.5
Trader	4.9	4.9	22.0	–	22.0	22	2.4	13.4	15.9
Driver	7.3	17.1	24.4	2.4	–	2.4	4.9	8.5	13.4
Civil servant	2.4	4.9	7.3	17.1	2.4	19.5	9.8	3.7	13.4
Artisan	4.9	12.2	17.1	7.3	7.3	14.6	6.1	9.8	15.9
**Ethnicity**									
Mole-Dagbani	4.9	14.6	19.5	4.9	4.9	9.8	4.9	8 (9.8	14.6
Akan	24.4	39.0	63.4	24.4	19.5	43.9	24.4	29.3	53.8
Ewe	7.3	2.4	9.8	7.3	12.2	19.5	7.3	7.3	14.6
Ga/Dangme	–	4.9	4.9	9.8	14.6	24.4	4.9	9.8	14.6
Nigerian	–	2.4	2.4	–	2.4	2.4	–	2.4	2.4
**Region of residence**									
Greater Accra	2.4	–	2.4	36.7	43.9	80.6	19.4	21.9	41.5
Central	2.4	2.4	4.9	7.3	4.9	12.2	4.9	3.6	8.5
Volta	–	–	–	–	2.4	2.4	–	1.2	1.2
Eastern	–	2.4	2.4	–	–	–	–	1.2	1.2
Ashanti	29.3	36.6	65.9	2.4	2.4	4.8	15.9	19.5	35.4
Northern	2.4	17.2	19.6	–	–	–	1.2	8.6	9.8
Upper East	2.4	–	2.4	–	–	–	1.2	–	1.2
Upper West	–	2.4	2.4	–	–	–	–	1.2	1.2
**CNCDs of Patients**									
Eye cancer	–	2.4	2.4	2.4	–	2.4	2.5	1.2	3.7
Prostate cancer	4.9	–	4.9	4.9	–	4.9	4.9	–	4.9
Breast cancer	–	2.4	2.4	–	–	–	–	1.2	1.2
Cervical cancer	–	–	–	–	2.4	2.4	–	1.2	1.2
Leukaemia	–	–	–	2.4	–	2.4	1.2	–	1.2
Asthma	2.4	–	2.4	2.4	4.9	7.3	2.4	2.4	4.9
Diabetes	2.4	7.3	9.8	–	2.4	2.4	1.2	4.9	6.2
Sickle cell	–	4.9	4.9	2.4	2.4	4.9	1.2	3.7	4.9
Stroke	4.9	9.8	14.6	4.9	7.3	12.2	4.9	8.5	13.4
Glaucoma	7.3	2.4	9.8	9.8	7.3	17.1	8.5	4.9	13.4
Chronic kidney disease	7.3	2.4	9.8	4.9	4.9	9.8	6.1	3.7	9.8
Chronic lung disease	–	4.9	4.9	–	2.4	2.4	–	3.7	3.7
Hypertension	2.4	4.9	7.3	–	7.3	7.3	1.2	6.1	7.3
Hypertension & Diabetes	2.4	7.3	9.8	2.4	4.9	7.3	2.4	6.1	8.5
Hypertension, Diabetes & stroke	–	2.4	2.4	–	–	–	–	1.2	1.2
Hypertension, Diabetes & glaucoma	–	2.4	2.4	_	–	–	–	1.2	1.2
Hypertension & Glaucoma	–	4.9	4.9	–	–	–	–	2.4	2.4
Hypertension & Chronic kidney disease	–	–	–	–	4.9	4.9	–	2.4	2.4
Hypertension & Prostate cancer	–	–	–	2.4)	–	2.4	1.2	–	1.2
Hypertension, Asthma & prostate cancer	–	–	–	2.4	–	2.4	1.2	–	1.2
Hypertension & stroke	2.4	2.4	2.4	2.4	2.4	4.9	2.4	2.4	4.9
Diabetes & breast cancer	–	–	2.4	–	2.4	2.4	–	1.2	1.2

### Thematic results

Table 4 presents the themes from our analysis. These were management practices by health professionals, self-management practices by patients, and challenges faced by health professional and patients in the management of CNCDs.

**Table 4:** Themes

Main themes	Sub-themes
General management practices by health professionals	General assessment of patients’ conditions
	• Checking of vital signs
	• Laboratory tests
	• History taking
	General education of patients
	• On the state of their conditions
	• On taking medications
	• On proper storage of the medicines
Specific management practices by health professionals	Based on specific CNCD presented
	• Treatment depends on the CNCD presented
	• Different medications and foods
	Based on the stage of the condition at presentation
	• Patients with early-stage presentation get life-saving interventions
	• Patients with late-stage presentation only get treatment to ease pain and suffering
Self-management practices by patients	Self-restrictions
	• Diet restrictions
	• Avoidance of triggers
	Exercise
	• Walking
	Personal first aid
	• Carrying out of warm water compression
	Use of anthropometric equipment to monitor health status
	• Monitoring of sugar level with a glucometer
Challenges in the management of CNCDs	Personal challenges of health professionals
	• Language barrier
	• Work-related stress emanating from heavy workload
	Institutional challenges of health professionals
	• Poor utility supply
	• Inadequate logistics
	• Inadequate staff
	• Inadequate motivation
	• Inadequate infrastructure
	• Inadequate in-service training
	Patient-related challenges
	• Financial challenges
	• Social challenges

### Health professionals’ experiences with CNCD management

Health professionals in our study had experiences with performing general and specific CNCD management practices. In healthcare practice, while general CNCD management practices usually entail services that are provided to the generality of patients irrespective of the conditions they present to a health facility, specific management practices relate to CNCD management services performed to ensure that tailored interventions are proffered to improve the health status of patients based on the CNCDs they present [37, 38]. In our study, the experiences of health professionals with the general management of CNCDs were mainly related to general assessments and education of patients on their conditions.

General assessments were done mainly by checking the vital signs of patients, conducting laboratory tests on them, and taking history regarding the CNCDs. The health professionals from both hospitals, for instance, noted that regarding checking of vital signs, the blood pressure, visual process, temperature, pulse, and respiration (TPR) of the patients were always checked irrespective of the CNCDs they presented. The checking of the vital signs enabled the health professionals to effectively manage the CNCDs. A nurse from KATH, for instance, had this to say regarding the checking of vital signs:

*Well! we usually check some vitals such as blood pressure and that runs for every patient, whether you are hypertensive, or not. This is important and has to be done so that we have a baseline with which to work and manage your condition.* We also take the weight and height for them.

***(Nurse, Female, 31 years)***

Laboratory testing also helped immensely to adequately understand the conditions presented by the patients. They argued that when the lab tests are conducted, they help to confirm the presence of the CNCDs in the patients. A nurse speaking on the tests conducted for patients in her department at KBTH noted:

*So, when they (patients) come, we screen to confirm what they have because some of them come and do not even know that they have other conditions … so we do everything (conduct a broad spectrum of tests) so that we are able to identify all possible conditions they have. So, we do the visual acuity test, gonioscopy…then we refer to the ophthalmologist.*

***(Nurse, Female, 40 years)***

History taking was also a key general CNCD management experience for the health professionals, as it gave them a clear understanding of disease onset and progression. History taking ensured effective management of the condition and a clear understanding of how they had been treating the conditions in the past.

*So, the doctor will take the history which is about how the thing started. You ask a bit about the family history. …we the family specialists take information on social life, the social setting… whether he is coming from a rich or poor family.*

***(Medical doctor, Male, 35 years)***

Patient education was a major general CNCD management experience espoused by the health professionals. Education is an essential component in the general practices involved in the management of CNCDs, as it ensures that patients adhere to management directives required for improvements in health status [39, 40]. The health professionals in our study carried out this service by educating the patients on the state of their conditions, how to take their medications, and how to properly store the medications. On education about the state of the CNCDs, the experiences of the health professionals entailed educating the patients to appreciate the stage of their conditions. In this regard, the health professionals intimated that sometimes, patients come to the health facilities as relapsed cases and, in such situations, education was done to orient them on the fact that the CNCD had become chronic. This information then enabled the patients to appreciate the stage their conditions had reached so that they give the conditions more attention and seriousness. A nurse from KATH also noted that they normally took the patients to medical doctors who educated them on their conditions.

*So, when they (patients) come, we educate and send them to the doctor…to tell them about their condition, and the stage that it is, whether it is advance or at the early stage.*

*(Nurse, Female, 30 years)*

The health professionals also usually educated the patients on the need to take their medications since that was the surest way of ensuring improved health status. They also educated the patients on the storage of their medications irrespective of their conditions. This is essential because, if medications are not stored under the right temperature, they have the propensity of losing their efficacy:

*We educate them on how to take the medications and also we inspect to make sure they follow the prescription. Those who do not follow the prescription are then educated again on the need to take them (medications) as expected*

***(Nurse, Female, 30 years)***

While the general management practices were performed by the health professionals irrespective of the CNCDs of patients, actual diagnosis and treatment depended mainly on the type of CNCD a patient presents. Medications given as well as foods to be eaten by patients varied based on the type of condition the patients presented. CNCD management practices carried out by the health professionals, for instance, varied when patients reported with either early-stage (normal) or late-stage (acute) conditions. Patients who reported early were given timely interventions including surgery to halt the level of deterioration of their conditions.

*So, after they’ve been diagnosed, those (glaucoma patients) who report early and, therefore, have their optic nerves to be healthy and their field vision hasn't gone that bad, can undergo surgery and then the eye will do better after the surgery.*

***(Nurse, Female, 34 years).***

Sometimes, however, the patients delayed at home before reporting their conditions. When that happens, their conditions deteriorate before they report. The health professionals, in such situations, only provided them with management options meant to ease pain and suffering but not necessarily to improve their conditions.

*…those (glaucoma patients) whose field vision is gone mainly because they came (reported) late and are just left with a tunnel vision, their optic nerve not that strong and their cornea not too good, the surgery will not be of benefit to them. So, what we do is to just give them medication to ease pain and suffering. That’s all.*

***(Nurse, Female, 34 years)***

The general CNCD management services provided by the health professionals reflect the services provided to patients in the general healthcare system of Ghana. They are, thus, not so different from services provided to non-CNCD patients. Irrespective of health conditions presented to hospitals in Ghana, general practices like history taking and checking of vital signs make it possible for health professionals to identify the genesis of the problem and to identify appropriate interventions that could be preferred. In a social system where 19% of women and 9% of men have no formal education [41], it is sometimes a daunting task for health professionals to conduct general assessments such as history taking, as some patients find it difficult to appropriately describe their disease progress. It is, however, on these general assessments that CNCD-specific treatment options are based and this makes their conduct very imperative. In the management of CNCDs, specific management practices usually follow the general management services provided by health professionals to patients. It is at this point that tailor-made services are provided to the patients based on the conditions they present. The experiences of the health professionals regarding their CNCD management practices, thus, reflect the situation in the Ghanaian context and across the globe.

### Patients’ experiences with CNCD management

The CNCD management experiences of patients were mainly related to their self-management practices. Self-management is a key component of optimal chronic disease care and results in effective and prompt management of CNCDs when implemented appropriately [42]. With self-management, patients are empowered to actively participate in their own management process [43]. In our study, self-management practices of patients entailed activities that the patients themselves put in place to manage the conditions especially when they were at home. We realised that the self-management practices of the patients were largely informed by self-management education given them by health professionals.

Exercise was an important self-management activity among patients, especially those living with hypertension, stroke, and diabetes. As a lot of the patients were 60 years and above and were quite weak and due to the debilitating effects of the CNCDs on their health, they mainly engaged in walking. A patient with hypertension for instance noted:

*With the exercise, I am able to do it. I am able to walk from here (KATH) to Tech (KNUST). Every day, I am able to walk about and I think it is good.*

*(Patient, Female, 65 years)*

Aside from walking, self-restrictions were practised by most of the patients. They mainly stopped eating certain diets they considered unhealthy. The diet restrictions focused on the type of foods to avoid and time to avoid eating. Some patients, for instance, stopped eating late and no longer ate some foods they were eating prior to being diagnosed with their conditions. The patients also avoided known triggers of their conditions. An asthmatic patient who usually experienced episodes of her asthma due to dust from dirty fans and louvre blades as well as unprescribed medications, for instance, tried to avoid such triggers:

*Well! for me, mostly my attack comes as a result of dust especially the ones on fan and louvres, so, I try to avoid them... I don’t take in any medicine aside from the asthma medication that has been prescribed for me because they can cause an attack.*

***(Patient, Male, 53 years)***

The patients also used anthropometric equipment to monitor their health status at home. Some diabetes patients, for instance, bought glucometers which they used to check their glucose level at home and always reported at the hospital whenever they realised higher than normal blood glucose levels. Some of the patients also used first aid to ensure that their conditions do not degenerate before they report to the health facilities. One of the patients with sickle cell disease, for instance, resorted to the use of warm water compression whenever she was about to have a complication.

*I do take my own first aid before it gets serious. Whenever I start feeling pains, I use warm water compression to treat myself, take in my drugs and then rest a bit to see what happens, if it still isn't working, I have to rush to the hospital, and see a doctor.*

***(Patient, Female, 43 years)***

As an LMIC, much of the efforts at managing CNCDs in Ghana, are in the hands of the patients. This is because the health system is usually overwhelmed with patients who are much more than health professionals (available to manage their conditions) and facilities to take care of all of them. Patients also spend most of their time at home and thus have to take charge of their own health while at home. The self-management practices realised in our study, to a large extent, reflect the popular avenues that patients use in the management of their conditions while at home. While patients use various forms of exercise, the main one realised in this study was walking, and this may be largely because the comparative majority of the study’s sample was above 50 years and cannot engage in the more vigorous exercises like jogging and running.

### CNCD management challenges of health professionals

Health professionals experienced a myriad of challenges in the management of CNCDs in our study. Chronic disease management has been noted as one of the most daunting challenges of healthcare systems across the globe, which drains patients, health professionals, and the overall health systems socially and economically [44, 45]. The challenges experienced by health professionals were sub-themed as personal, institutional, and patient-related challenges. The patient-related challenges have been triangulated with challenges experienced by the patients themselves under the sub-heading “Patients’ challenges with CNCD management”.

### Personal challenges of health professionals

Personal challenges of the health professionals comprised language barrier and work-related stress emanating from the heavy workload. Regarding the language barrier, most of the health professionals noted that due to variations in the ethnic backgrounds of the CNCDs patients, they (the health professionals) were not able to speak/understand some of the patients’ languages.

*Sometimes, there are some things you need to tell the patient or to know her exact need…But you will be talking to the patient and the patient can’t understand what you are saying. She will be talking to you and you can’t also understand what he or she is saying… So, the language barrier is a huge hindrance*

***(Nurse, Female, 26 years)***

There were, however, instances where they called on colleagues who understood the languages to explain procedures, instructions, and conditions to the patients. Those who could not get colleagues to interpret resorted to the use of signs to communicate with the patients.

Most of the health professionals also experienced work-related stress which they noted to have emanated from a heavy workload. They reported experiencing work-related stress due to the high number of patients they attend to daily.

*Yes! there is (work-related) stress because the patients become many and at times, the treatment takes a long time and when you are treating patients at times, the treatment will not work well because maybe the cancer changed its course so we have to change the type of chemo drugs they are given.*

***(Nurse, Female, 58 years)***

### Institutional challenges of health professionals

The main institutional challenges were poor utility supply, inadequate logistics, staff, motivation, infrastructure, and in-service training. Regarding poor utility supply, the health professionals regularly experienced water shortages and intermittent electricity outages which interfered with CNCD management activities.

*The water is not flowing. It can take like 5 to 6 days and it won’t flow. Right now, just go to the washroom and see. You’ll cry… And this place too is a female ward… basic handwashing technique is even poor because, how to get water is even a problem.*

*(Nurse, Male, 28 years)*

With regards to inadequate logistics, health professionals from both hospitals noted that CNCD management was constrained by the inadequacy of blood pressure (bp) monitors, prescription cards, tissue paper, continuation sheets, and equipment essential for the assessment, monitoring, and performing of management procedures were either inadequate or not available in some of the departments. CNCD management was also constrained by inadequate beds and stockout of medications and other consumables, and these challenges were more prevalent at KATH than KBTH. For instance, there are times that pharmacies lacked some medications which the health professionals felt should have been available:

*Yea! there are some drugs that the pharmacy doesn’t have, so you have to go outside and buy them. So in this case, if the money is not available that means we can’t provide that management for the patient…So, the medicines unavailability especially when they are needed is a major challenge over here.*

***(Nurse, Female, 26 years)***

Due to lack of accommodation, many of the health professionals stayed very far from the hospital before coming to work daily. By the time they got to work, they were already exhausted. When they closed from work too, they had to enter commercial vehicles in the night (especially when running afternoon and evening shifts) to get home. This routine, therefore, negatively affected their productivity at work. We also found that from both facilities, infrastructure was inadequate. The health professionals explained that there was not enough space for service delivery and staff accommodation. A female health professional from KATH for instance noted that narrated their ordeal regarding the space in which her outfit operated. She said:

*Hmm, yes! Our clinic is very small. Sometimes, when you call the patients they don’t get anywhere to sit. The patients too are many so if they can expand the place a little, it would help. When the patients are crowded on you like that it makes working difficult and uneasy.*

***(Nurse, Female, 30 years)***

Even though several CNCD cases are reported at the various units/departments of the hospitals either on a referral or review basis daily, there was an inadequacy of health professionals to attend to such cases. This, therefore, negatively affected CNCD treatment timelines, quality of care, and overall management outcomes of patients. Many of the health professionals attributed the staff shortage to the lack of financial clearance to employ trained health professionals by the state.

*Staff shortage…we are understaffed. We are not getting new staff because there is no financial clearance for them to be employed so it is a problem…currently, people are getting to know more about physiotherapy...so you have more patients trooping in and wanting to be seen…so it’s a problem.*

***(Physiotherapist, Female, 31 years)***

Inadequacy of motivation was also a major challenge experienced by the health professionals in our study. While the health professionals expected to be given bonuses, end-of-year packages, and awards for working hard and attending to patients with CNCDs diligently, such motivations were generally not forthcoming. At KBTH, for instance, some of the health professionals noted that in the past, there were weekly and monthly bonuses that were given to them. About a year before our study, however, management of the facility stopped giving them such bonuses. At the end of the calendar year (in December), they were usually given some Christmas packages by the hospital management. The health professionals, however, complained that such packages were usually not adequate. For others, however, there was no such thing as staff motivation at their respective department/facilities as they had not experienced them since joining the hospital as workers.

*As for motivation, I don’t want to talk about it, so you see that I never talk about staff motivation? It will not be forthcoming so even if you say it, you just talk in vain.*

*(Nurse, Female, 35 years)*

In-service training was also not regularly organised for the staff. Some of the health professionals from both hospitals noted that workshops and conferences were not regularly organised to equip them with skills in the latest technologies and strategies for CNCD care. They, therefore, still use procedures that they considered outdated in the management of CNCDs.

*We don’t get that kind of regular in-service training or regular updates. So, that becomes a huge challenge. We still use the very out-moded procedures and all that…if you get specialists to organise programmes for us, it will help, but that doesn’t happen often.*

***(Nurse, Male, 31 years)***

Ghana is a multilingual country where there are over 80 local languages spoken [46]. Even though the official language is English, people usually prefer speaking in their local languages. The country also has a quite high illiteracy level, which makes it difficult for a significant proportion of the population to speak the English language. There are, therefore, challenges with language when it comes to inter-ethnic exchanges/interactions. The findings regarding the language barrier, therefore, posit with the Ghanaian context appropriately. As a developing country, Ghana has a health system that is mainly inundated by a myriad of challenges that militate against the effective delivery of health services. While health facilities strive to provide top-up training in the form of workshops and conferences, these are usually not enough due to challenges with funding for such programmes. The challenge of inadequate in-service training is, therefore, a systemic one. As a result of the growing population of Ghana, the generally higher life expectancy rates, as well as the availability of refined foods, coupled with poor eating habits, the number of people requiring CNCD services keeps increasing. This, however, comes at the backdrop that the country faces grave human resource and infrastructural deficits needed to meet the growing demand for CNCD services. The challenges of limited space and inadequacy of health professionals, as mentioned by health professionals in our study, therefore, reflect the systemic challenges inherent in the country’s health system.

### Patients’ challenges with CNCD management

The onset of a CNCD and its management usually have grave socio-economic consequences for patients and their families [47]. In the present study, we found that patients’ challenges with the management of their CNCDs were mainly financial and social.

### Financial challenges of patients

Financial challenges were, for instance, intimated by both health professionals and patients to include the inability of the patients to afford the costs involved in managing their CNCDs. Medications and surgeries that need to be conducted are usually too expensive for the patients to afford. Many patients also experience challenges getting transportation fare needed to attend scheduled reviews:

*Sometimes, it’s financial constraints. Even the inability to cater for their transportation. So sometimes, they fail to come for review. So, I can say it’s about financial constraints*.

***(Physiotherapist, Female, 27 years)***

*The medications are expensive. Sometimes, I inject insulin and when it gets finished, I can go and buy one medication like GHȼ (GH₵ 100 [GHȼ: The Ghana Cedi, is Ghana’s official currency). At times I am not able to buy and inject. So right now, my financial state is really down.*

***(Patient, Male, 54 years)***

Even though the medications were expensive, some were not covered by the country’s social health insurance policy (the National Health Insurance Scheme [NHIS]) which would have made them more affordable to the patients:

*All the drugs are expensive but are not on health insurance except some cases of breast cancer that we have insurance support for…not part of the health insurance medicines list...we have one medicine here and it is about GHȼ 6,000 but, it is not covered by the NHIS.*

**(*Nurse, Female, 34 years)***

Some of the patients were also not able to purchase anthropometric equipment for monitoring health at home due to financial challenges. A stroke patient from KATH, for instance, had this to say:

*Yes! I have challenges with the machine that help me to walk. When I went to Atonsu Agogo, they (the health facility) had some device that is used in doing walking exercise. I would have preferred to have some to use at home but I don’t have enough money to buy it. It really disturbs me.*

***(Patient, Female, 50 years)***

### Social challenges of patients

The main social challenge we identified among patients was the nature of their job limiting treatment adherence. We found that due to their respective occupations, some of the patients were not able to go for their reviews on time. Some of them were also not able to take their medications on time.

*I’m a teacher and I have to be in school at some particular times while am supposed to be here (at the hospital) too…The timing is one major problem I’m facing and it makes it difficult for me to come for review sometimes as indicated by the health workers.*

*–KATH, Patient, Female, 26 years*

The health professionals also expressed concern that some patients usually took herbal medications while on the orthodox management for their conditions. Some also replaced the orthodox medications they were given with herbal medications completely. A nurse from KATH had this to say:

*When they go home, they are told that they can take other drugs to heal them as well. They end up adding herbal medicines to the ones they have been given at the hospital. It makes it difficult for them to get better when that happens*

*( Nurse, Female, 30 years)*

A nurse from KBTH also gave instances where patients abandoned their medications to depend on herbal ones only to return with aggravated conditions. She posited:

*Yes, I know some patients who went for alternative care and came back in wheelchairs. There was this patient whom I knew was doing well and there was progress in his condition. Suddenly, I was not seeing the patient. So later, the patient came back in a wheelchair and according to the relative of the patient, they took some herbal concoctions and all that. Some also end up at prayer camps but come back worse off because they stop using the medicines we give them.*

*(Nurse, Female, 35 years)*

In Ghana, a lot of the working population earn below the minimum wage [48], which points to the high rates of poverty within the adult population. When an individual becomes incapacitated in terms of being unable to work and earn an income due to a CNCD, it exerts a lot of financial constraints on the individual. Considering that medications and surgical procedures carried out in the management of CNCDs are quite expensive, it becomes difficult for them to afford. This, therefore, explains why the patients generally could not afford the cost of managing their CNCDs. With about 80% of Ghanaians relying on traditional medicine for primary health care [49], there is clearly a high level of trust in traditional medicine among the Ghanaian populace. This, therefore, explains why some patients abandoned their orthodox medications to focus on herbal ones.

## Discussion

This was a qualitative study that explored the experiences of patients and health professionals with CNCD management at KATH and KBTH in Ghana. We found that the experiences of health professionals regarding CNCD management entailed the general assessments and education they provided to the patients irrespective of the conditions presented and the CNCD-specific care they provided based on the type and stage of the conditions. Patients’ experiences with CNCD management were mainly in the form of self-management practices they carried out mainly at home. We also found that a myriad of challenges including language barrier, self-reported stress emanating from a heavy workload on the part of health professionals, and inability to afford the cost of managing the conditions on the part of patients, militated against effective management of CNCDs.

The findings of the current study where the CNCD management experiences of CNCDs involved general and specific management practices by health professionals and self-management practices by patients, thus, support the conceptual framework’s postulations that clinical care by health professionals and patients’ roles through self-management are the major means through which CNCDs are managed [50]. Rea et al. [51], in this regard, also argued that management of CNCDs focuses on high-quality care and patient empowerment for self-management. We observed patient empowerment in our study as involving the self-management education provided by the health professionals on the state of the conditions and taking of medications. In Ghana, this is essential, as it ensures that patients are better positioned to take care of their own health while at home. It also supplements conventional patient education by supporting patients with CNCDs to live the best possible quality of life. The finding regarding self-management education also corroborates the conceptual framework’s position that effective chronic care entails patient self-management empowerment through self-management education [23, 24].

Self-management practices by patients were found in this study as an essential component of CNCD management. In Ghana, patients spend most of their time at home before returning to health facilities for reviews. The need to self-manage their conditions is, thus, the key to ensuring that they do not relapse or experience adverse health outcomes. This confirms our conceptual framework’s postulation that self-management practices by patients constitute core components in the management of CNCDs [52] where the patients use coherent strategies (or not coherent) with clinicians’ recommendation [43].

Persons living with CNCDs mainly grapple with the social, psychological, and physical strains which include dealing with signs and symptoms, negative emotional impacts, disability, difficult lifestyle adjustments, complex medication regimens, and access to useful medical care [50]. The conceptual framework of the present study, therefore, recognises that to support patients in dealing with the vicissitudes of living with the CNCDs, effective management should have a properly systematised healthcare delivery system [50]. The institutional challenges of poor utility supply and inadequate logistics, staffing, motivation, infrastructure, and in-service training which were realised in the present study, therefore, point to the possible deficiencies that impede the ability of the health system to effectively handle CNCDs in the country [53]. Our findings regarding the institutional challenges also resonate with a WHO report which opined that the management of CNCDs exerts great indirect and direct costs on health system budgets [54]. To assemble nationwide responses, most countries establish governmental units in their respective Ministries of Health to address the spiralling burden of CNCDs. However, most health systems do not have operational nationwide plans such as chronic care management policies in place [54].

The institutional-level challenges observed in our study could also be attributed to the inadequate political commitment by states to accelerate the priority given to non-communicable diseases in the national health and development agenda [54]. The inadequate political commitment, according to the WHO [55], is demonstrated by the low levels of priority given to CNCDs in health development strategies and the minimal funding allocated to the control and prevention of such diseases. Thus, national plans and policies for the control and prevention of CNCDs are always underfunded.

Our findings where patients had financial challenges in affording the cost of management, in getting transportation fare for review, and in acquiring anthropometric equipment for monitoring the conditions at home confirm findings from previous studies that CNCDs have serious socio-economic consequences for patients through increased individual and household impoverishment [56, 57]. Participants in our study attributed patients’ inability to afford the cost of managing their conditions to the fact that even though expensive, management of CNCDs was generally not covered by the NHIS which would have made it cheaper. The NHIS is Ghana’s social health insurance policy established in the year 2003 to ease the financial burden from accessing healthcare particularly on the poor [58]. While the NHIS covers about 95% of the disease burden of the country and most out-patient and in-patient services including surgeries, emergency care, accommodation at the wards of health facilities and drugs listed on the medicines list of the scheme [59, 60], the fact that the drugs needed by the patients in the present study were not covered by the NHIS, thus, implies they are not on the medicines list. The plethora of challenges faced in the management of CNCDs reflects the systemic lapses inherent in the health system of Ghana [61] and which militate against the possible achievement of the SDG 3.4 target of reducing by one third, premature mortality from CNCDs through prevention and treatment by the year 2030 [7, 62].

### Strengths and limitations

This was the first hospital-based study aimed at understanding the management of CNCDs from the perspective of patients and health professionals in Ghana. The involvement of both patients and health professionals helped to present a balanced scope of CNCDs management. Even though data were collected from two hospitals, the study has a nationwide scope in that patients recruited were from all over the country. Data collection was done through in-depth interviews at the respective hospitals which could have introduced response bias on the part of the participants. The use of purposive sampling introduced the possibility of selection bias on the part of the data collectors. The possible implication of these biases is that all issues regarding the management of CNCDs might not have been entirely covered in the current study.

## Conclusions

We found that a myriad of challenges inhibits the effective management of CNCDs among patients. The challenges militate against progress towards meeting the SDG 3 of ensuring healthy lives and promoting wellbeing for all at all ages and specifically reducing, by one third, premature mortality from CNCDs by the year 2030. Our findings also imply that even though the hospitals put a lot of resources in place to improve the management of CNCDs, there is still a lot of work and efforts required in ensuring improved health outcomes for persons living with CNCDs. We recommend that the Ministry of Health and the Ghana Health Service, in collaboration with the management of the respective hospitals, should ensure adequate provision of logistics and infrastructure, recruitment of more health professionals, regular in-service training, improved utility supply, and adequate staff motivation. The Ministry of Health and the National Health Insurance Authority should review the NHIS to cover the management of all CNCDs. The Ministry of Health should also develop and implement a chronic care management policy to give more priority to the management of CNCDs.

## Supplementary Information

**Additional file 1.** Consolidated criteria for reporting qualitative research (COREQ)

**Additional file 2.** Data collection instrument

## Data Availability

All relevant data are within the manuscript and its Supporting Information files. Any further requests regarding the data used for this study could be made through the corresponding author.
