# Supplementary material for: Management of chronic non-communicable diseases in Ghana: a qualitative study using the chronic care model
Source: BMC Public Health. 2021 Jun 11;21:1120. doi: 10.1186/s12889-021-11170-4 (PMC8196497; doi:10.1186/s12889-021-11170-4)
Supplement: Supplementary file 1 — Additional file 1. Consolidated criteria for reporting qualitative research (COREQ) [file 12889_2021_11170_MOESM1_ESM.pdf]

Management of chronic non-communicable diseases in Ghana: A qualitative study

Hubert Amu[1,2*], Eugene Kofuor Maafo Darteh[2], Elvis Enowbeyang Tarkang[1], Akwasi Kumi-Kyereme[2]

[1]Department of Population and Behavioural Sciences, School of Public Health, University of Health and Allied Sciences, Hohoe, Ghana

[2]Department of Population and Health, University of Cape Coast, Cape Coast, Ghana

Consolidated criteria for reporting qualitative research (COREQ): a 32-item checklist for interviews and focus groups

ALLISON TONG[1,2], PETER SAINSBURY[1,3] AND JONATHAN CRAIG[1,2]

[1] School of Public Health, University of Sydney, NSW 2006, Australia, [2] Centre for Kidney Research, The Children's Hospital at Westmead,
NSW 2145, Australia, and [3]Population Health, Sydney South West Area Health Service, NSW 2170, Australia

Abstract

Background. Qualitative research explores complex phenomena encountered by clinicians, health care providers, policy makers and consumers. Although partial checklists are available, no consolidated reporting framework exists for any type of qualitative design.

Objective. To develop a checklist for explicit and comprehensive reporting of qualitative studies (indepth interviews and focus groups).

Methods. We performed a comprehensive search in Cochrane and Campbell Protocols, Medline, CINAHL, systematic reviews of qualitative studies, author or reviewer guidelines of major medical journals and reference lists of relevant publications for existing checklists used to assess qualitative studies. Seventy-six items from 22 checklists were compiled into a comprehensive list. All items were grouped into three domains: (i) research team and reflexivity, (ii) study design and (iii) data analysis and reporting. Duplicate items and those that were ambiguous, too broadly defined and impractical to assess were removed.

Results. Items most frequently included in the checklists related to sampling method, setting for data collection, method of data collection, respondent validation of findings, method of recording data, description of the derivation of themes and inclusion of supporting quotations. We grouped all items into three domains: (i) research team and reflexivity, (ii) study design and (iii) data analysis and reporting.

Conclusions. The criteria included in COREQ, a 32-item checklist, can help researchers to report important aspects of the research team, study methods, context of the study, findings, analysis and interpretations. Keywords: focus groups, interviews, qualitative research, research design

Qualitative research explores complex phenomena encountered by clinicians, health care providers, policy makers and consumers in health care. Poorly designed studies and inadequate reporting can lead to inappropriate application of qualitative research in decision-making, health care, health policy and future research.

Formal reporting guidelines have been developed for randomized controlled trials (CONSORT) [1], diagnostic test studies (STARD), meta-analysis of RCTs (QUOROM) [2], observational studies (STROBE) [3] and meta-analyses of observational studies (MOOSE) [4]. These aim to improve the quality of reporting these study types and allow readers to better understand the design, conduct, analysis and findings of published studies. This process allows users of published research to be more fuller informed when they critically appraise studies relevant to each checklist and decide upon applicability of research findings to their local settings. Empiric studies have shown that the use of the CONSORT statement is associated with improvements in the quality of reports of randomized controlled trials [5]. Systematic reviews of qualitative research almost always show that key aspects of study design are not reported, and so there is a clear need for a CONSORT-equivalent for qualitative research [6].

The Uniform Requirements for Manuscripts Submitted to Biomedical Journals published by the International Committee of Medical Journal Editors (ICMJE) do not provide reporting guidelines for qualitative studies. Of all the mainstream biomedical journals (Fig. 1), only the British Medical Journal (BMJ) has criteria for reviewing qualitative

Downloaded from https://academic.oup.com/intqhc/article-abstract/19/6/349/1791966 by guest on 14 April 2020

research. However, the guidelines for authors specifically record that the checklist is not routinely used. In addition, the checklist is not comprehensive and does not provide specific guidance to assess some of the criteria. Although checklists for critical appraisal are available for qualitative research, there is no widely endorsed reporting framework for any type of qualitative research [7].

We have developed a formal reporting checklist for in-depth interviews and focus groups, the most common methods for data collection in qualitative health research.

Address reprint requests to: Allison Tong, Centre for Kidney Research, The Children's Hospital at Westmead, NSW 2145, Australia. Tel: þ61-2-9845-1482; Fax: þ61-2-9845-1491; E-mail: allisont@health.usyd.edu.au, allisont@chw.edu.au

The Author 2007. Published by Oxford University Press on behalf of International Society for Quality in Health Care; all rights reserved

Downloaded from https://academic.oup.com/intqhc/article-abstract/19/6/349/1791966 by guest on 14 April 2020

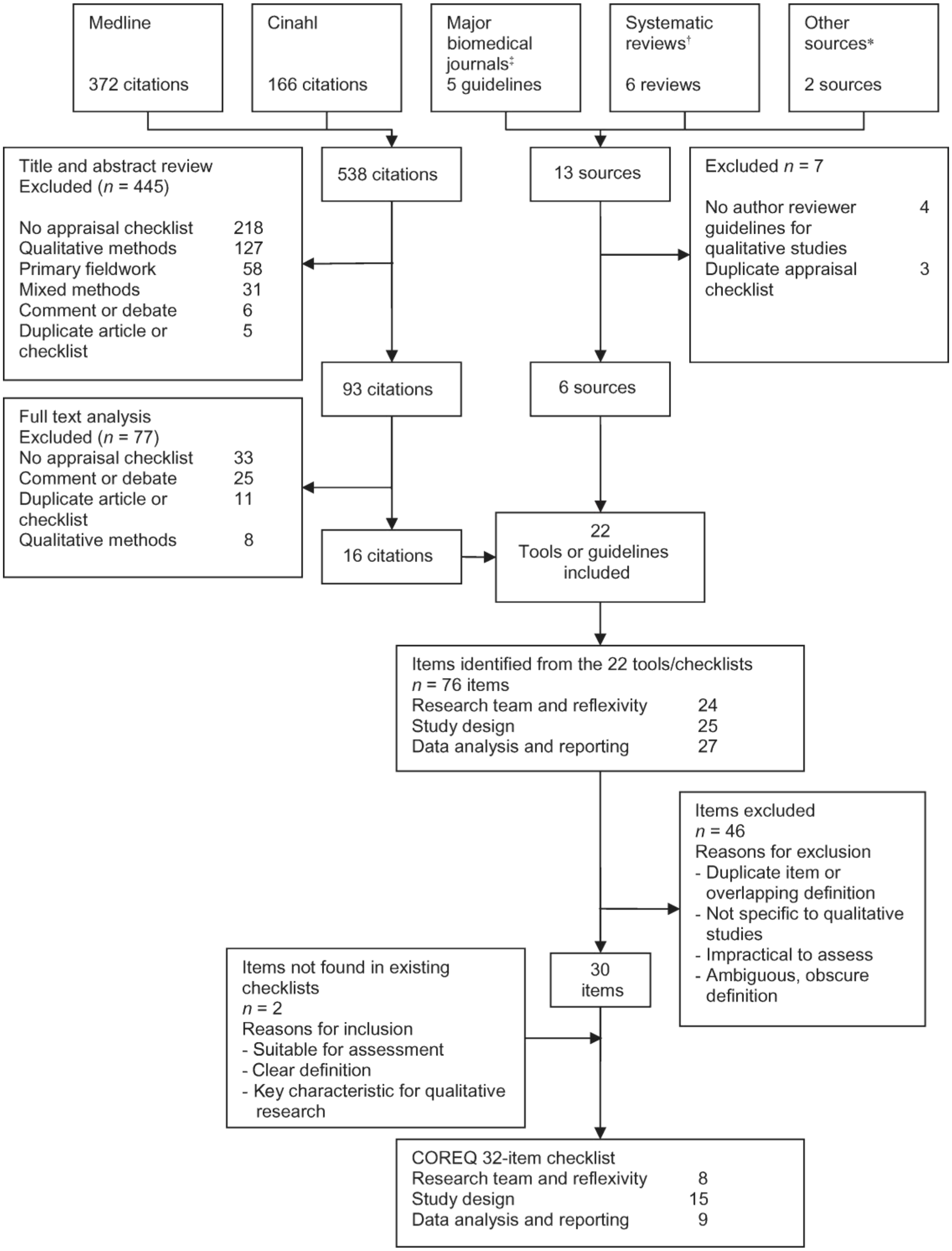

Figure 1 Development of the COREQ Checklist. *References [26, 27], †References [6, 28–32], ‡Author and reviewer guidelines provided by BMJ, JAMA, Lancet, Annals of Internal Medicine, NEJM.

These two methods are particularly useful for eliciting patient and consumer priorities and needs to improve the quality of health care [8]. The checklist aims to promote complete and transparent reporting among researchers and indirectly improve the rigor, comprehensiveness and credibility of interview and focus-group studies.

Basic definitions

Qualitative studies use non-quantitative methods to contribute new knowledge and to provide new perspectives in health care. Although qualitative research encompasses a broad range of study methods, most qualitative research publications in health care describe the use of interviews and focus groups [8].

Interviews

In-depth and semi-structured interviews explore the experiences of participants and the meanings they attribute to them. Researchers encourage participants to talk about issues pertinent to the research question by asking open-ended questions, usually in one-to-one interviews. The interviewer might re-word, re-order or clarify the questions to further investigate topics introduced by the respondent. In qualitative health research, in-depth interviews are often used to study the experiences and meanings of disease, and to explore personal and sensitive themes. They can also help to identify potentially modifiable factors for improving health care [9].

Focus groups

Focus groups are semi-structured discussions with groups of 4–12 people that aim to explore a specific set of issues [10]. Moderators often commence the focus group by asking broad questions about the topic of interest, before asking the focal questions. Although participants individually answer the facilitator's questions, they are encouraged to talk and interact with each other [11]. This technique is built on the notion that the group interaction encourages respondents to explore and clarify individual and shared perspectives [12]. Focus groups are used to explore views on health issues, programs, interventions and research.

Methods

Development of a checklist

Search strategy. We performed a comprehensive search for published checklists used to assess or review qualitative studies, and guidelines for reporting qualitative studies in: Medline (1966—Week 1 April 2006), CINAHL (1982— Week 3 April 2006), Cochrane and Campbell protocols, systematic reviews of qualitative studies, author or reviewer guidelines of major medical journals and reference lists of relevant publications. We identified the terms used to index the relevant articles already in our possession and performed a broad search using those search terms. The electronic databases were searched using terms and text words for research (standards), health services research (standards) and qualitative studies (evaluation). Duplicate checklists and detailed instructions for conducting and analysing qualitative studies were excluded.

Data extraction. From each of the included publications, we extracted all criteria for assessing or reporting qualitative studies. Seventy-six items from 22 checklists were compiled into a comprehensive list. We recorded the frequency of each item across all the publications. Items most frequently included in the checklists related to sampling method, setting for data collection, method of data collection, respondent

validation of findings, method of recording data, description of the derivation of themes and inclusion of supporting quotations. We grouped all items into three domains: (i) research team and reflexivity, (ii) study design and (iii) data analysis and reporting. (see Tables 2–4)

Within each domain we simplified all relevant items by removing duplicates and those that were ambiguous, too broadly defined, not specific to qualitative research, or impractical to assess. Where necessary, the remaining items were rephrased for clarity. Based upon consensus among the authors, two new items that were considered relevant for reporting qualitative research were added. The two new items were identifying the authors who conducted the interview or focus group and reporting the presence of non-participants during the interview or focus group. The COREQ checklist for explicit and comprehensive reporting of qualitative studies consists of 32 criteria, with a descriptor to supplement each item (Table 1).

COREQ: content and rationale (see Tables 1)

Domain 1: research team and reflexivity

(i) Personal characteristics: Qualitative researchers closely engage with the research process and participants and are therefore unable to completely avoid personal bias. Instead researchers should recognize and clarify for readers their identity, credentials, occupation, gender, experience and training. Subsequently this improves the credibility of the findings by giving readers the ability to assess how these factors might have influenced the researchers' observations and interpretations [13–15].

(ii) Relationship with participants: The relationship andextent of interaction between the researcher and their participants should be described as it can have an effect on the participants' responses and also on the researchers' understanding of the phenomena [16]. For example, a clinician– researcher may have a deep understanding of patients' issues but their involvement in patient care may inhibit frank discussion with patient–participants when patients believe that their responses will affect their treatment. For transparency, the investigator should identify and state their assumptions and personal interests in the research topic.

Domain 2: study design

(i) Theoretical framework: Researchers should clarify thetheoretical frameworks underpinning their study so readers can understand how the researchers explored their research questions and aims. Theoretical frameworks in qualitative research include: grounded theory, to build theories from the data; ethnography, to understand the culture of groups with shared characteristics; phenomenology, to describe the meaning and significance of experiences; discourse analysis, to analyse linguistic expression; and content analysis, to systematically organize data into a structured format [10].

Downloaded from https://academic.oup.com/intqhc/article-abstract/19/6/349/1791966 by guest on 14 April 2020

sampling is used which involves selecting participants who share particular characteristics and have the

Table 1

1. Interviewer/facilitator Which author/s conducted the interview or focus group?
2. Credentials What were the researcher's credentials? E.g. PhD, MD
3. Occupation What was their occupation at the time of the study?
4. Gender Was the researcher male or female?
5. Experience and training What experience or training did the researcher have?

Relationship with participants

6. Relationship established Was a relationship established prior to study commencement?
7. Participant knowledge of the What did the participants know about the researcher? e.g. personal goals, reasons for doing the interviewer research
8. Interviewer characteristics What characteristics were reported about the interviewer/facilitator? e.g. Bias, assumptions, reasons and interests in the research topic

Domain 2: study design

Theoretical framework

9. Methodological orientation and What methodological orientation was stated to underpin the study? e.g. grounded theory,
Theory discourse analysis, ethnography, phenomenology, content analysis

Participant selection

10. Sampling How were participants selected? e.g. purposive, convenience, consecutive, snowball
11. Method of approach How were participants approached? e.g. face-to-face, telephone, mail, email
12. Sample size How many participants were in the study?
13. Non-participation How many people refused to participate or dropped out? Reasons?

Setting

14. Setting of data collection Where was the data collected? e.g. home, clinic, workplace
15. Presence of non-participants Was anyone else present besides the participants and researchers?
16. Description of sample What are the important characteristics of the sample? e.g. demographic data, date

Data collection

17. Interview guide Were questions, prompts, guides provided by the authors? Was it pilot tested?
18. Repeat interviews Were repeat interviews carried out? If yes, how many?
19. Audio/visual recording Did the research use audio or visual recording to collect the data?
20. Field notes Were field notes made during and/or after the interview or focus group?
21. Duration What was the duration of the interviews or focus group?
22. Data saturation Was data saturation discussed?
23. Transcripts returned Were transcripts returned to participants for comment and/or correction?

Consolidated criteria for reporting qualitative studies (COREQ): 32-item checklist

No Item Guide questions/description

Domain 1: Research team and reflexivity

Personal Characteristics

Domain 3: analysis and findingsz

Data

24. Number of data coders How many data coders coded the data?
25. Description of the coding tree Did authors provide a description of the coding tree?
26. Derivation of themes Were themes identified in advance or derived from the data?
27. Software What software, if applicable, was used to manage the data?
28. Participant checking Did participants provide feedback on the findings?

Reporting

analysis

29. Quotations presented Were participant quotations presented to illustrate the themes / findings? Was each quotation identified? e.g. participant number
30. Data and findings consistent Was there consistency between the data presented and the findings?
31. Clarity of major themes Were major themes clearly presented in the findings?
32. Clarity of minor themes Is there a description of diverse cases or discussion of minor themes?

(ii) Participant selection: Researchers should report howparticipants were selected. Usually purposive

potential to provide rich, relevant and diverse data pertinent to the research question

Downloaded from https://academic.oup.com/intqhc/article-abstract/19/6/349/1791966 by guest on 14 April 2020

[13, 17]. Convenience sampling is less optimal because it may fail to capture important perspectives from difficultto-reach people [16]. Rigorous attempts to recruit participants and reasons for non-participation should be stated to reduce the likelihood of making unsupported statements [18].

Table 2 Items included in 22 published checklists: Research team and reflexivity domain

Item	References																					
	[26][a]	[27][a]	[6][b]	[28][b]	[32][b]	[13]	[15]	[14]	[17]	[33]	[34]	[35]	[16]	[19]	[36]	[7]	[37]	[23]	[38]	[39]	[22]	BMJ
Research team and reflexivity																						
Nature of relationship between the researcher and participants		†		†	†		†		†						†				†			
Examination of role, bias, influence	†				†	†	†	†							†						†	
Description of role		†		†					†					†						†		
Identity of the interviewer		†		†		†					†		†		†							
Continued and prolonged engagement		†				†							†						†			
Response to events	†					†	†	†														
Prior assumptions and experience		†						†									†			†		
Professional status		†					†								†							
Journal, record of personal experience		†								†				†								
Effects of research on researcher		†				†																
Qualifications		†													†							
Training of the interviewer / facilitator			†		†																	
Expertise demonstrated		†																	†			
Perception of research at inception								†						†								
Age							†															
Gender							†															
Social class							†															
Reasons for conducting study		†																				
Sufficient contact													†									
Too close to participants													†									
Empathy																	†					
Distance between researcher and participants							†															
Background								†														
Familiarity with setting																					†	

[a]Other publications, [b]Systematic review of qualitative studies; BMJ, British Medical Journal—editor's checklist for appraising qualitative research); †, item included in the checklist.

Downloaded from https://academic.oup.com/intqhc/article-abstract/19/6/349/1791966 by guest on 14 April 2020

Table 3 Items included in 22 published checklists: Study design

Item	References
	[26][1] [27][a] [6][b] [28] [b] [32][b] [13] [15] [14] [17] [33] [34] [35] [16] [19] [36] [7] [37] [23] [38] [39] [22] BMJ
Study design	
Methodological orientation, ontological or epistemological basis	† † † † † † † † †
Sampling—convenience, purposive	† † † † † † † † † † † † † † † † † †
Setting	† † † † † † †
Characteristics and description of sample	† † † † † †
Reasons for participant selection	† † † † †
Non-participation	† † † †
Inclusion and exclusion, criteria	† † † †
Identity of the person responsible for recruitment	† † † †
Sample size	† † † † †
Method of approach	† † †
Description of explanation of research to participants	† † †
Level and type of participation	†
Method of data collection, e.g. focus group, in-depth interview	† † † † † † † † † † † † † † †
Audio and visual recording	† † † † † † † † † † † †
Transcripts	† † † † † † † † †

[1] Other publications, [b]Systematic review of qualitative studies; BMJ, British Medical Journal—editor's checklist for appraising qualitative research; †, item included in the checklist.

Downloaded from https://academic.oup.com/intqhc/article-abstract/19/6/349/1791966 by guest on 14 April 2020

Setting and location	†	†							†	†		†	†	†					†						†	†
Saturation of data	†	†	†								†		†			†	†							†		
Use of a topic guide, tools, questions	†	†	†											†						†	†	†				
Field notes					†	†	†	†													†					†
Changes and modifications	†	†							†	†											†			†		
Duration of interview, focus group				†							†			†									†			
Sensitive to participant language and views				†											†			†								
Number of interviews, focus groups				†							†															
Time span																								†		
Time and resources available to the study				†																						

Table 4 Items included in 22 published checklists: Analysis and reporting

Item	References																					
	[26][a]	[27][a]	[6][b]	[28][b]	[32][b]	[13]	[15]	[14]	[17]	[33]	[34]	[35]	[16]	[19]	[36]	[7]	[37]	[23]	[38]	[39]	[22]	BMJ

Respondent validation	†	†	†		†		†		†				†				†	†	†	†		
Limitations and generalizability	†			†			†		†		†		†					†				
Triangulation	†			†	†	†	†	†	†					†			†		†			
Original data, quotation		†	†	†	†			†			†			†		†				†	†	†
Derivation of themes explicit	†	†	†	†	†		†				†								†			†
Contradictory, diverse, negative cases	†			†			†			†				†					†			†
Number of data analysts	†	†	†			†			†			†							†			†
In-depth description of analysis	†			†				†			†			†							†	
Sufficient supporting data presented	†			†			†				†					†						
Data, interpretation and conclusions linked and integrated		†		†								†		†						†		
Retain context of data		†					†							†					†			
Explicit findings, presented clearly	†			†					†													
Outside checks													†					†				
Software used		†				†													†			†
Discussion both for and against the researchers' arguments	†			†																		
Development of theories, explanations		†					†			†		†										
Numerical data		†									†							†				†
Coding tree or coding system		†					†												†		†	
Inter-observer reliability		†									†										†	
Sufficient insight into meaning/perceptions of participants		†								†												
Reasons for selection of data to support findings		†			†																	
New insight		†						†														
Results interpreted in credible, innovative way									†													
Eliminate other theories													†									
Range of views														†								
Distinguish between researcher and participant voices								†														
Proportion of data taken into account														†								

[a]Other publications, [b]Systematic review of qualitative studies; BMJ, British Medical Journal—editor's checklist for appraising qualitative research, †, item included in the checklist.

Downloaded from https://academic.oup.com/intqhc/article-abstract/19/6/349/1791966 by guest on 14 April 2020

Researchers should report the sample size of their study to enable readers to assess the diversity of perspectives included.

(iii) Setting: Researchers should describe the context inwhich the data were collected because it illuminates why participants responded in a particular way. For instance, participants might be more reserved and feel disempowered talking in a hospital setting. The presence of non-participants during interviews or focus groups should be reported as this can also affect the opinions expressed by participants. For example, parent interviewees might be reluctant to talk on sensitive topics if their children are present. Participant characteristics, such as basic demographic data, should be reported so readers can consider the relevance of the findings and interpretations to their own situation. This also allows readers to assess whether perspectives from different groups were explored and compared, such as patients and health care providers [13, 19].

(iv) Data collection: The questions and prompts used indata collection should be provided to enhance the readers' understanding of the researcher's focus and to give readers the ability to assess whether participants were encouraged to openly convey their viewpoints. Researchers should also report whether repeat interviews were conducted as this can influence the rapport developed between the researcher and participants and affect the richness of data obtained. The method of recording the participants' words should be reported. Generally, audio recording and transcription more accurately reflect the participants' views than contemporaneous researcher notes, more so if participants checked their own transcript for accuracy [19–21]. Reasons for not audio recording should be provided. In addition, field notes maintain contextual details and non-verbal expressions for data analysis and interpretation [19, 22]. Duration of the interview or focus group should be reported as this affects the amount of data obtained. Researchers should also clarify whether participants were recruited until no new relevant knowledge was being obtained from new participants (data saturation) [23, 24].

Domain 3: analysis and findings

(i) Data analysis: Specifying the use of multiple coders orother methods of researcher triangulation can indicate a broader and more complex understanding of the phenomenon. The credibility of the findings can be assessed if the process of coding (selecting significant sections from participant statements), and the derivation and identification of themes are made explicit. Descriptions of coding and memoing demonstrate how the researchers perceived, examined and developed their understanding of the data [17, 19]. Researchers sometimes use software packages to assist with storage, searching and coding of qualitative data. In addition, obtaining feedback from participants on the research findings adds validity to the researcher's interpretations by ensuring that the participants' own meanings and perspectives are represented and not curtailed by the researchers' own agenda and knowledge [23].

(ii) Reporting: If supporting quotations are provided, researchers should include quotations from different

participants to add transparency and trustworthiness to their findings and interpretations of the data [17]. Readers should be able to assess the consistency between the data presented and the study findings, including the both major and minor themes. Summary findings, interpretations and theories generated should be clearly presented in qualitative research publications.

Discussion

The COREQ checklist was developed to promote explicit and comprehensive reporting of qualitative studies (interviews and focus groups). The checklist consists of items specific to reporting qualitative studies and precludes generic criteria that are applicable to all types of research reports. COREQ is a comprehensive checklist that covers necessary components of study design, which should be reported. The criteria included in the checklist can help researchers to report important aspects of the research team, study methods, context of the study, findings, analysis and interpretations.

At present, we acknowledge there is no empiric basis that shows that the introduction of COREQ will improve the quality of reporting of qualitative research. However this is no different than when CONSORT, QUOROM and other reporting checklists were introduced. Subsequent research has shown that these checklists have improved the quality of reporting of study types relevant to each checklist [5, 25], and we believe that the effect of COREQ is likely to be similar. Despite differences in the objectives and methods of quantitative and qualitative methods, the underlying aim of transparency in research methods and, at the least, the theoretical possibility of the reader being able to duplicate the study methods should be the aims of both methodological approaches. There is a perception among research funding agencies, clinicians and policy makers, that qualitative research is 'second class' research. Initiatives like COREQ are designed to encourage improvement in the quality of reporting of qualitative studies, which will indirectly lead to improved conduct, and greater recognition of qualitative research as inherently equal scientific endeavor compared with quantitative research that is used to assess the quality and safety of health care. We invite readers to comment on COREQ to improve the checklist.

Downloaded from https://academic.oup.com/intqhc/article-abstract/19/6/349/1791966 by guest on 14 April 2020

References

1. Moher D, Schulz KF, Altman D. The CONSORT statement: revised recommendations for improving the quality of reports of parallel-group randomized trials. JAMA 2001;285:1987–91.
2. Moher D, Cook DJ, Eastwood S et al. Improving the quality of reports of meta-analyses of randomised controlled trials: the QUOROM statement. Quality of Reporting of Meta-analyses. Lancet 1999;354:1896–900.
3. STROBE Statement: Strengthening the reporting of observational studies in epidemiology. http://www.strobe-statement.org/Checkliste.html
4. Stroup DF, Berlin JA, Morton SC et al. Meta-analysis of observational studies in epidemiology: a proposal for reporting. Meta-analysis Of Observational Studies in Epidemiology (MOOSE) group. JAMA 2000;283:2008–12.
5. Moher D, Jones A, Lepage L. Use of the CONSORT Statement and quality of reports of randomized trials. A comparative before-and-after evaluation. JAMA 2001;285:1992–5.
6. Mills E, Jadad AR, Ross C et al. Systematic review of qualitative studies exploring parental beliefs and attitudes toward childhood vaccination identified common barriers to vaccination. J Clin Epidemiol 2005;58:1081–8.
7. Knafl KA, Howard NJ. Interpreting and reporting qualitativeresearch. Res Nurs Health 1984;7:7–14.
8. Sofaer S. Qualitative research methods. Int J Qual Health Care 2002;14:329–36.
9. Wright EB, Holcombe C, Salmon P. Doctor’s communicationof trust, care, and respect in breast cancer: qualitative study. BMJ 2004;328:864–8.
10. Liamputtong P, Ezzy D. Qualitative Research Methods. 2nd edn. Melbourne, Victoria: Oxford University Press, 2005.
11. Krueger RA, Casey MA. Focus Groups. A Practical Guide for Applied Research. Thousand Oaks CA: Sage Publications, 2000.
12. Morgan DL. Focus Groups as Qualitative Research. Newbury Park, California: Sage, 1988.
13. Giacomini MK, Cook DJ. Users’ guides to the medical literature XXIII. Qualitative research in health care. A. Are the results of the study valid? JAMA 2000;284:357–62.
14. Malterud K. Qualitative research:standards challenges guidelines. Lancet 2001;358:483–8.
15. Mays N, Pope C. Qualitative research in health care: assessingquality in qualitative research. BMJ 2000;320:50–2.
16. Elder NC, William L. Reading and evaluating qualitative research studies. J Fam Pract 1995;41:279–85.
17. Cote L, Turgeon J. Appraising qualitative research articles inmedicine and medical education. Med Teach 2005;27:71–5.
18. Altheide D, Johnson J. Criteria for assessing interpretive validityin qualitative research. In Denzin N, Lincoln Y (eds). Handbook of Qualitative Research. Thousand Oaks CA: Sage Publications, 1994.
19. Fossey E, Harvey C, McDermott F et al. Understanding and evaluating qualitative research. Aust N Z J Psychiatry 2002;36:717–32.
20. Seale C, Silverman S. Ensuring rigour in qualitative research.Eur J Public Health 1997;7:379–84.
21. Scheff T. Single case analysis in the health sciences. Eur J Public Health 1995;5:72–4.
22. Bluff R. Evaluating qualitative research. Br J Midwifery 1997;5:232–5.

23. Popay J, Rogers A, Williams G. Rationale and standards for thesystematic review of qualitative literature in health services research. Qual Health Res 1998;8:341–51.
24. Blumer H. Critiques of Research, in the Social Sciences. New Brunswick, NJ: Transaction Books, 1979.
25. Delaney A, Bagshaw SM, Ferland A et al. A systematic evaluation of the quality of meta-anlyses in the critical care literature. Crit Care 2005;9:575–82.
26. Critical Skills Appraisal Programme (CASP) 10 Questions tohelp you make sense of qualitative research: Milton Keynes Primary Care Trust, 2002.
27. Spencer L, Ritchie J, Lewis J et al. Quality in Qualitative Evaluation: A Framework for Assessing Research Evidence. London: Cabinet Office. Government Chief Social Researcher’s Office, 2003.
28. Campbell R, Pound P, Pope C et al. Evaluating meta-ethnography: a synthesis of qualitative research on lay experience of diabetes and diabetes care. Soc Sci Med 2003;56:671–84.
29. Feder GS, Hutson M, Ramsay I et al. Women exposed to intimate partner violence: expectations and experiences when they encounter health care professionals: a meta-analysis of qualitative studies. Arch Intern Med 2006;166:22–37.
30. Pound P, Britten N, Morgan M et al. Resisting medicines: a synthesis of qualitative studies of medicine taking. Soc Sci Med 2005;61:133–55.
31. Smith LK, Pope C, Botha JL. Patients’ help-seeking experiencesand delay in cancer presentation: a qualitative synthesis. Lancet 2005;366:825–31.
32. Walter FM, Emery J, Braithwaite D et al. Lay understanding of familial risk of common chronic diseases: a systematic review and synthesis of qualitative research. Ann Fam Med 2004; 2:583–94.
33. Inui TS, Frankel RM. Evaluating the quality of qualitativeresearch: a proposal pro-term. J Gen Intern Med 1991;6:485–6.
34. Boulton M, Fitzpatrick R, Swinburn C. Qualitative research inhealth care: II A structured review and evaluation of studies. J Eval Clin Pract 1996;2:171–9.
35. Dixon-Woods M, Shaw RL, Agarwal S et al. The problem of appraising qualitative research. Qual Saf Health Care 2004;13:223–5.
36. Hoddinott P, Pill R. A review of recently published qualitativeresearch in general practice. More methodological questions than answers? Fam Pract 1997;14:313–9.
37. Kuzel AJ, Engel JD, Addison RB et al. Desirable features of qualitative research. Fam Pract Res J 1994;14:369–78.
38. Treloar C, Champness S, Simpson PL et al. Critical appraisal checklist for qualitative research studies. Indian J Pediatr 2000;67:347–51.

Downloaded from https://academic.oup.com/intqhc/article-abstract/19/6/349/1791966 by guest on 14 April 2020

39. Cesario S, Morin K, Santa-Donato A. Evaluating the level ofevidence in qualitative research. J Obstet Gynecol Neonatal Nurs 2001;31:708–14.

Accepted for publication 7 July 2007
