# Supplementary material for: Management of chronic non-communicable diseases in Ghana: a qualitative study using the chronic care model
Source: BMC Public Health. 2021 Jun 11;21:1120. doi: 10.1186/s12889-021-11170-4 (PMC8196497; doi:10.1186/s12889-021-11170-4)
Supplement: Supplementary file 2 — Additional file 2. Data collection instrument [file 12889_2021_11170_MOESM2_ESM.pdf]

Management of chronic non-communicable diseases in Ghana: A qualitative study

Hubert Amu[1,2*], Eugene Kofuor Maafo Darteh[2], Elvis Enowbeyang Tarkang[1], Akwasi Kumi-Kyereme[2]

[1]Department of Population and Behavioural Sciences, School of Public Health, University of Health and Allied Sciences, Hohoe, Ghana

[2]Department of Population and Health, University of Cape Coast, Cape Coast, Ghana

Data Collection Instruments

S2_A. In-depth interview guide for patients

Interview number: ________________

Date of interview (DD/MM/YY): ____/___/______

Time of interview: Start ___End____

Interviewer: ________________

SECTION A: Socio-demographic Characteristics of participants

1. Are you a male or female ______________
2. How old are you? ______________
3. What is your marital status? ______________
4. What religion do you practice? ______________
5. What is your ethnicity? ______________
6. What is your educational level? ______________
7. Where do you reside (region and town)? ______________
8. What is your occupation? ______________
9. What chronic non-communicable disease(s) have you been diagnosed with?
10. How long have you been living with this condition(s)? (Probe for number of years/months participant has been living with the disease/year in which the participant was diagnosed with the chronic non-communicable disease).
11. How long have you been attending this facility for the management of your condition(s)? (Probe for number of months/years of attending the specific facility

SECTION B: Management of chronic non-communicable diseases

12. How central are you in the management of your condition by health professionals in this facility? (Probe for the frequency, and instances where participant was involved or consulted in planning and implementing management strategies).
13. How do you personally manage your condition? (Probe for self-care or self-regulatory activities such as taking one's medicine on time, use of physical indicator devices such as scale, sphygmomanometer, and diet monitor, going for reviews as scheduled, adherence to diet, exercise, and other management strategies)

SECTION C: Challenges associated with the management of chronic noncommunicable diseases by patients

14. What are the major challenges you encounter in management of your condition? (Probe for financial and social constraints [lack of social support and negative perceptions of family and friends towards condition which negatively influence their level of support for the patient in managing the condition]).
15. Are there other challenges you face in the management of your condition which we have not spoken about?

Thank you.

S2_B. In-depth interview guide for health professionals

Interview number: _______________

Date of interview (DD/MM/YY): ____/____/_____

Time of interview: Start ___End____

Interviewer: _______________

SECTION A: Socio-demographic Characteristics of participants

1. Are you a male or female _______________
2. How old are you? _______________
3. What is your marital status? _______________
4. What religion do you practice? _______________
5. What is your ethnicity? _______________
6. What is your educational level? _______________
7. What is your job title? _______________
8. For how long have you been practising? _______________ 9. How long have you been practising in this facility? _______________

SECTION B: Management of chronic non-communicable diseases

10. Which conditions do you generally manage in this facility?
11. How do you normally manage patients with the chronic non-communicable diseases in this facility? (Probe for processes and procedures used to cater for patients; also probe for the main medication/suggestions on self-management options that are provided to patients)

SECTION C: Challenges associated with the management of chronic noncommunicable diseases by the health professionals

12. What personal challenges do you face in the management of patients with chronic non-communicable diseases? (Probe for language barrier, lack of training on management of the diseases, work-related stress, time and workloads, poor interpersonal relationship with clients and other staff, etc.)
13. What institutional challenges do you encounter in the management of patients with chronic non-communicable diseases? (Probe for lack of logistics such as testing devices and drugs, institutional support, and motivation)
14. What patient-related challenges do you face in the management of patients with chronic non-communicable diseases? (Probe for non-adherence of patients to management directives such as exercise, diet, medicines, reporting for review etc.; poor financial strength of patients, seeking alternative treatment from diverse sources, poor attitude of patients towards their own management and health professionals etc.)
15. What other challenges do you encounter during the management of clients with chronic non-communicable diseases?
16. How do these challenges affect management of chronic non-communicable disease services rendered in this facility?
17. In what ways can these challenges be addressed? (By facility, by government, by patients, and by other stakeholders in health).

Thank You.
